# Construction Notes

**Published:** 1897-04-03

**Authors:** 


### CONSTRUCTION NOTES.

**Epidemic Hospital at Inverurie, N.B.**

The fever hospital which has been erected at Inverurie has now been brought into use. The hospital has been built by the Garioch District Committee and Inverurie Town Council, and it is intended to serve the whole of the district, including the burgh of Inverurie. The situation is an open one, and the building was designed by Messrs. Jenkins and Mard, architects, of Aberdeen. It consists of a ground floor and attics, and is in the form of the letter T, with the head of the letter to the west. The building is divided into two entirely separate houses, entered by different doors. The smaller house, which occupies the foot of the letter, includes two wards, which can be thrown into one, and which are at present fitted with three beds each. There is also a small kitchen and fair-sized room in the attic, as well as lavatory accommodation in this part of the building. The other part is far the larger, and occupies the whole of the west, or cross wing, as well as part of the main structure. The west wing includes a large and small ward, which can be thrown together by opening the folding doors. In the large ward there are at present seven beds, while in the small one there are three beds. Additional beds, however, can be put up in case of necessity. In the main structure are the kitchen, matron's room, bath-room, &c., and in the attics are several bed-rooms. The cost of the construction was about £2,000.

